# Literary Note

**Published:** 1907-03

**Authors:** 


### LITERARY NOTE.

A few years ago the New Orleans Picayune published a most remarkable story of a man who was lying in a condition closely resembling death in the house and under the care of the eminent oculist, Dr. Clancey Courteneay and an equally eminent physician, Dr. Richard, both of New Orleans. Some months after, the paper again refered to the case, the subject having then been in a comatose state for nearly six months. By this time the facts had become known to the leading physicians of the city, who were much puzzled and were watching for the outcome with great interest. The case was discussed then and is still referred to as The Strange Case of Dr. Bruno.

Dr. Courteneay has since died, leaving among his manuscripts a full account of the affair, and his heir and executor, Dr. F. E. Daniel, has given it to the world. A stranger story was never penned. Indeed, it would be quite incredible if its truth were not attested by such high authority.

Dr. Bruno, who, after years of study, tried the experiment upon himself of suspending animation without destroying life, was a fellow student of Dr. Courteneay's at Heidelberg, and they became intimate frieds. He was quite successful in the experiment, and lay in a comatose state, without food or water, for six months, but was killed by a shock at the very moment of his awakening. He had left his story in a manuscript and Dr. Courteneay gives it in full.

Aside from the wonderful experiment, Dr. Bruno's life was curious and eventful, containing a romance which the reader will eagerly follow.

The Strange Case of Dr. Bruno is just published by the The Guarantee Publishing Co., New York.

